# Supplementary material for: Hybrid Models and Biological Model Reduction with PyDSTool
Source: PLoS Comput Biol. 2012 Aug 9;8(8):e1002628. doi: 10.1371/journal.pcbi.1002628 (PMC3415397; doi:10.1371/journal.pcbi.1002628)
Supplement: Text S4 — Complete source code for the PyDSTool package (version 0.88.120504). Includes API documentation and help files linking to web pages. This file is identical to the current public release on Sourceforge.net. (ZIP) [file pcbi.1002628.s004.zip › PyDSTool/html/PyDSTool.Toolbox.optimizers.criterion.composite_criteria-module.html]

xml version="1.0" encoding="ascii"?


PyDSTool.Toolbox.optimizers.criterion.composite\_criteria


| Home | Trees | Indices | Help | PyDSTool |
| --- | --- | --- | --- | --- |

Package PyDSTool ::
Package Toolbox ::
Package optimizers ::
Package criterion ::
Module composite\_criteria

[hide private]

[frames] | no frames]

# Module composite\_criteria

source code

Composite criteria allow to use several criteria together, with and/or
composition


|  |  |
| --- | --- |
| Classes | [hide private] |

OrComposition  
Compose several criteria with an or rule

AndComposition  
Compose several criteria with an and rule


|  |  |
| --- | --- |
| Functions | [hide private] |

|  |  |
| --- | --- |
| all(iterable) | source code |

|  |  |
| --- | --- |
| any(iterable) | source code |

| Home | Trees | Indices | Help | PyDSTool |
| --- | --- | --- | --- | --- |

|  |  |
| --- | --- |
| Generated by Epydoc 3.0.1 on Fri May 4 15:24:05 2012 | http://epydoc.sourceforge.net |
